# *Equid alphaherpesvirus 1* from Italian Horses: Evaluation of the Variability of the *ORF30*, *ORF33*, *ORF34* and *ORF68* Genes

**DOI:** 10.3390/v11090851

**Published:** 2019-09-13

**Authors:** Silvia Preziuso, Micaela Sgorbini, Paola Marmorini, Vincenzo Cuteri

**Affiliations:** 1 School of Biosciences and Veterinary Medicine, University of Camerino, Via Circonvallazione 93/95, 62024 Matelica (MC), Italy; vincenzo.cuteri@unicam.it; 2 Department of Veterinary Sciences, University of Pisa, San Piero a Grado, 56122 Pisa, Italy; micaela.sgorbini@unipi.it; 3 Private Practitioner, 52100 Arezzo, Italy; ninnaro@tiscali.it

**Keywords:** *Equid alphaherpesvirus 1*, horse, PCR, sequencing, *ORF30*, *ORF33*, *ORF34*, *ORF68*

## Abstract

*Equid alphaherpesvirus 1* (EHV-1) is an important pathogen of horses. It is spread worldwide and causes significant economic losses. The *ORF33* gene has a conserved region that is often used as target in diagnostic PCR protocols. Single nucleotide point (SNP) mutations in *ORF30* are usually used to distinguish between neuropathogenic and non-neuropathogenic genotypes. An *ORF68* SNP-based scheme has been used for grouping different isolates. Recently, the highest number of variable sites in EHV-1 from the UK has been found in ORF34. In this study, EHV-1 positive samples from Italian horses with a history of abortion were investigated by amplifying and sequencing the *ORF30*, *ORF33*, *ORF34* and *ORF68* genes. Most animals were infected by the neuropathogenic type A2254G. A 118 bp deletion was found at nucleotide positions 701–818 of the *ORF68* gene, making impossible to assign the samples to a known group. Sequencing of the *ORF34* gene with a newly designed nested PCR showed new SNPs. Analysis of these sequences and of those obtained from genetic databases allowed the identification of at least 12 groups. These data add depth to the knowledge of EHV-1 genotypes circulating in Italy.

## 1. Introduction

*Equid alphaherpesvirus 1* (EHV-1) is a DNA virus belonging to the genus *Varicellovirus* in the family *Herpesviridae*, subfamily *Alphaherpesvirinae*. Infection with EHV-1 is included in the World Organization for Animal Health (OIE) List [1] because it causes abortions, respiratory disease and neurological disease, with significant economic losses in the equine industry worldwide. The genome contains 76 open reading frames (ORFs) predicted to encode functional proteins [2]. The *ORF33* gene, encoding the glycoprotein B (gB), possesses a conserved region that is frequently used as target for diagnostic PCR protocols [3]. The *ORF30* gene, which encodes the DNA polymerase gene, is considered a marker of pathogenicity because the potential to cause neuropathogenicity is significantly higher in EHV-1 strains that carry a single nucleotide point (SNP) mutation in this gene [4]. The A to G mutation at nucleotide position 2254 of ORF30 causes a substitution of asparagine (N) to aspartic acid (D) at amino acid position 752 in the catalytic subunit of the viral DNA polymerase. EHV-1 N752 is referred to as a non-neuropathogenic genotype, and D752 as a neuropathogenic genotype. This single amino acid mutation causes replication to a higher level and longer viremia in experimentally infected horses, when compared to animals infected with EHV-1 lacking this particular mutation [5,6]. Comparison between the genomic sequences of the neuropathogenic strain Ab4 and of the non-neuropathogenic strain V592 showed that the major amino acid residue differences were in a membrane-associated virion component encoded by the *ORF68* gene. In particular, a region of about 600 bp in *ORF68* was particularly polymorphic, and therefore it was tentatively adopted as a marker system for efficiently grouping the isolates into six groups [4]. This method was subsequently used for comparing isolates from different geographical regions [7,8,9,10,11,12]. Recently a Multi-locus analysis approach, based on sequencing heterologous regions in 26 open reading frames, proved a more comprehensive method of strain typing than only *ORF68* sequencing. [12]. An extensive study, covering at least 80% of the genome for each of 78 EHV-1 isolated between 1982 and 2016 mostly in UK, demonstrated that the V32 protein, encoded by the *ORF34* gene, was the most variable in this viral collection [13]. Considering that genotyping studies on EHV-1 circulating in Italy are very limited [12,14] and that a few sequences of Italian EHV-1 are available in public genetic sequence databases (GenBank and European Nucleotide Archive-ENA, last access 1st July 2019), the aim of this study was to investigate the variability of the *ORF30*, *ORF33*, *ORF34* and *ORF68* genes of EHV-1 positive samples collected from Italian horses with a history of abortion, in comparison with sequences available in genetic databases and the bibliography.

## 2. Materials and Methods

### 2.1. Samples

DNA archival samples collected from Italian horses in 2008–2010 and in 2017–2019 were available for this study. Samples from mares that aborted, from aborted fetuses and from a recumbent horse had been collected by veterinarians in sterile containers and had been sent to the laboratory within 24 h in an airtight box containing cold accumulators for the diagnosis of beta-hemolytic C-streptococci infection [15,16]. Some horses were regularly vaccinated against EHV-1 (Table 1). DNA had been obtained immediately after samples arrival to the laboratory by a commercial kit (Genomic DNA isolation kit, Norgen Biotek Corp., Thorold, ON, Canada) following the manufacturer’s instructions. Two-hundred DNA samples stored at –20 °C were selected for this study and were tested by nested PCR (nPCR) to detect EHV-1 and EHV-4 [17]. Briefly, primers FC2 (5′-CTTGTGAGATCT AACCGCAC-3′) and RC (5′-GGGTATAGAGCTTTCATGGG-3′) targeting a common sequence of EHV-1 and EHV-4 were used in a first PCR. Subsequently, nPCRs were performed to amplify a 188 bp sequence of EHV-1 with primers FC3 (5′-ATACGATCACATCCAATCCC-3′) and R1 (5′-GCGTTATAGCTATCACGTCC-3′) or to amplify a 677 bp sequence of EHV-4 with primers FC3 and R4 (5′-CCTGCATAATGACAGCAGTG-3′). First round PCR mixture included 50 μL 2× Taq PCR Master Mix (Qiagen, Hilden, Germany), 25 pmol each primer (FC2 and RC), 2 μL DNA, and PCR grade water up to 50 μL final volume. Second round PCRs included 50 μL 2× Taq PCR Master Mix (Qiagen), 25 pmol each primer (FC3 and R1 or FC3 and R4), 5 μL of the product of the first round PCR, and PCR grade water up to 50 μL final volume. Amplification conditions of both first and second round PCRs were 94 °C for 5 min, 40 cycles at 94 °C for 1 min, 60 °C for 1 min and 72 °C for 1 min, with a final extension at 72 °C for 7 min followed by refrigeration at 4 °C. PCR products (10 μL each) were visualized in 1.5% agarose gel. Twenty positive samples collected from twelve different stables located in three different geographical regions (Marche, Tuscany and Veneto) or collected from the same stable but in different years were selected for sequencing and analysis of the genes *ORF30*, *ORF33*, *ORF34* and *ORF68* (Table 1).

### 2.2. PCR and Sequencing

All primers used in PCR and sequencing reactions are reported in Table 2. Nested PCR protocols were used because single PCR (sPCR) showed limited sensitivity. A 256 bp product of the *ORF30* gene including the polymorphic site A2254G, which is suspected to determine the viral pathogenicity [6], was obtained by nPCR [18]. After alignment of all EHV-1 *ORF33*, *ORF34* and *ORF68* sequences available in GenBank and in ENA, the most variable sequences were selected to be amplified by nPCR. A new set of primers (FC2int/RCint) was designed to amplify a 940 bp sequence of the *ORF33* gene by nPCR using the products obtained by first PCR with primers FC2/RC [17] as template.

After preliminary unsuccessful tests with primers used to amplify the *ORF68* gene by sPCR [10,12], new primers were designed (68p1-Fe/68p1-Re and 68p2-Fi/68p2-Ri) to obtain by nPCR a 774 bp product including the sequence used for grouping the isolates [4]. Studies on the *ORF34* gene are limited and primers to amplify this gene are not described. Two pairs of primers were designed to amplify the entire ORF34 gene by nPCR (1058F/1893R and 1090Fi/1784Ri). The primers are complementary to a terminal sequence of the *ORF33* gene and an initial sequence of the *ORF35* gene. All new primers were designed with Primer3Plus [19] and were tested by Nucleotide BLAST to evaluate their similarity with EHV-1 or with other unspecific sequences. The PCR protocols were optimized by using an EHV-1 isolate as positive control and an EHV-1-negative equine sample as negative control. All new nPCR protocols were dedicated only for sequencing and have been not tested for diagnostic purposes.

After the optimization of the amplification protocols, the mixture of the first PCRs included 25 µL 2× Taq PCR Master Mix (Qiagen), 500 nM each primer (F8/R2, FC2/RC, 1058F/1893R, or 68p1-Fe/68p1-Re), 2 µL DNA, and PCR grade water up to 50 µL final volume. PCR conditions were 94 °C for 5 min, 45 cycles of 94 °C for 1 min, appropriate annealing temperature (Table 2) for 1 min, 72 °C for 1 min, and a final extension of 72 °C for 7 min. The second PCRs were carried out with the same amplification conditions but with primers F7/R3, FC2int/RCint, 1090Fi/ 1784Ri or 68p2-Fi/68p2-Ri; 2 µL of the first PCR products were used as template for nPCRs with primers F7/R3 or 1090Fi/ 1784Ri and 5 µL of the first PCR products were used as template for nPCRs with primers FC2int/RCint or 68p2-Fi/68p2-Ri. PCR products were visualized in 1.0% agarose gel and positive samples were submitted to an external laboratory for sequencing by Sanger method (BMR Genomics, Padua, Italy). Both sense and antisense strands were sequenced. If discordant results were obtained or if new SNPs were observed in the sequences, PCRs and sequencing were repeated. To limit the number of identical sequences included in GenBank, only representative sequences were deposited (Accession numbers MN226968-MN226990, Table S1).

### 2.3. Sequence Analysis

Nucleotide sequences were manually checked and edited with the program BioEdit 7.0.5 [20]. Sequences were aligned by MUSCLE [21]. Phylogenetic tree with representative ORF34 sequences were inferred with the program MEGA 7.0.21 [22]. The best-fitting nucleotide substitution model were estimated; Kimura 2-parameter model with gamma-distributed rates among sites was used for *ORF33* analysis and Tamura-3-parameter model was used for *ORF34* analysis, both with bootstrap values based on 1000 repetitions. Phylogeny was estimated by both the Neighbor-Joining algorithm (NJ) and the maximum likelihood (ML) method.

## 3. Results

A total of 20 sequences of *ORF30* and *ORF34*, 10 sequences of *ORF33* and seven sequences of *ORF68* were suitable for analysis. The sequences were aligned with those available in public databases and SNPs were investigated.

Two out of 20 samples had adenine (A) in position 2254 of the *ORF30* gene and 18 had guanine (G), showing that most animals were infected by the EHV-1 neuropathogenic variant N752. Other SNPs were not present in the 256 bp sequence analyzed in comparison with the reference strain Ab4.

The nucleotide sequence of the EHV-1 Ab4 strain (GenBank Accession number AY665713.1) [2] served as a basis also for the comparison of nucleotide changes in the *ORF33*, *ORF34* and *ORF68* genes.

**Figure 1:**
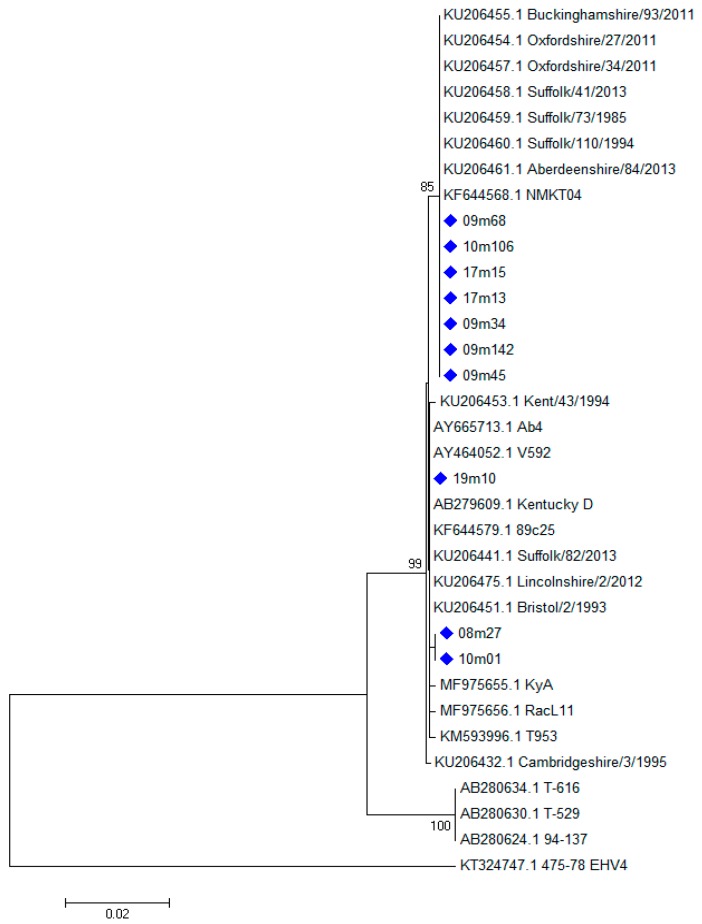
Phylogenetic tree showing the evolutionary history of the *ORF33* sequences from nt 1525 to nt 2409 (Neighbor-Joining method with bootstrap test with 1000 replicates). The evolutionary distances were computed using the Kimura 2-parameter method and are in the units of the number of base substitutions per site. The rate variation among sites was modeled with a gamma distribution (shape parameter = 5). Sequences obtained in this study are marked with a diamond (♦).

A limited variability of the *ORF33* sequences was observed (Figure 1). A non-synonymous SNP was found in seven out of 10 samples at position A1526T (Table 3), corresponding to the amino acidic substitution N509I. The same seven samples showed also a synonymous mutation at position G2391A. Samples 08m27 and 10m01 showed a change at A1531G, corresponding to the amino acidic substitution N601D. Furthermore, longer sequences were obtained by first round PCR from samples 09m34, 09m45, 09m142 and 19m10. Sample 19m10 showed three non-synonymous changes at I810V, I838P and A861G, while no further changes were found in the other three samples (Table 3).

**Figure 2:**
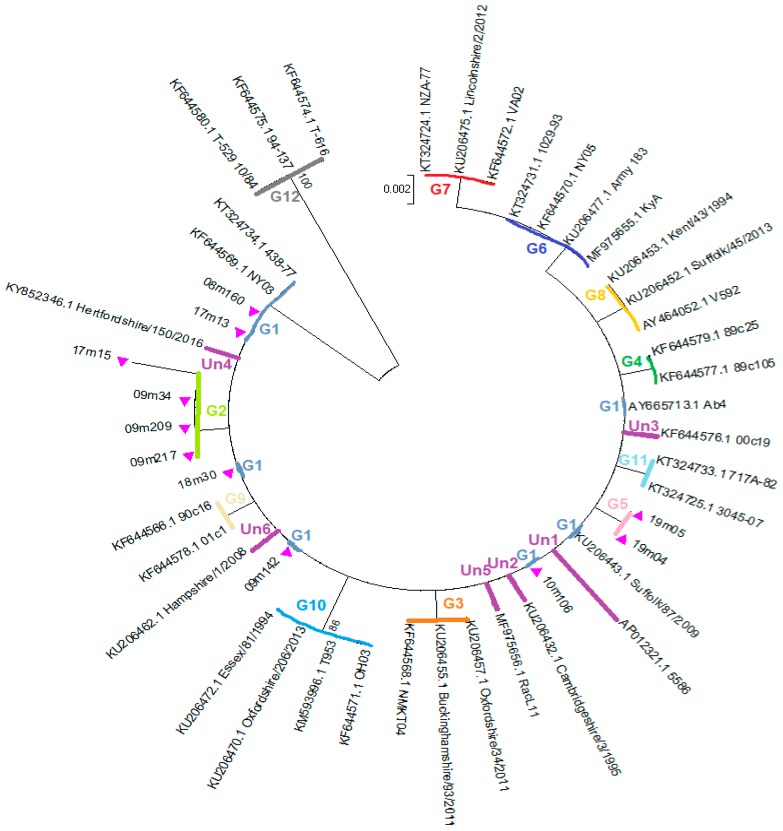
The evolutionary history of the *ORF34* sequences was obtained by Neighbor-Joining method with bootstrap test with 1000 replicates. The evolutionary distances were computed using the Tamura 3-parameter method and are in the units of the number of base substitutions per site. Sequences obtained in this study are marked with a triangle ▲. The letter “G” followed by a number indicates the number of the group where the sequences are located. The letters “Un” followed by a number indicates the sequences not located in any Group.

The new nPCR protocol to amplify the *ORF34* gene showed a high sensitivity because all samples that resulted positive according to the diagnostic nPCR method of Wang et al. [17] were also positive by this nPCR and all samples provided a high amount of PCR products, which resulted sufficient and suitable for sequencing. Analysis of the 714 bp *ORF34* nucleotide sequences obtained in this study showed a synonymous mutation at T60C in four samples, one of which showed also two non-synonymous changes at C380T and T410C, corresponding to changes at amino acid positions T127I and V137A respectively. Two samples showed a non-synonymous SNP at C149T, while the other 14 samples showed the same *ORF34* sequence as the reference strain Ab4 (Table 4). A total of 114 *ORF34* gene sequences were obtained from GenBank database and were aligned with the sequences obtained in this study. Sequence analysis suggested that all the EHV-1 *ORF34* gene sequences available in GenBank and all sequences obtained in this study could be categorized tentatively into twelve groups (Table 4). A simplified tree including only selected sequences representative of each observed nucleotide variation is reported in Figure 2.

A group was generated when at least two sequences were identical and showed the same variations at the same positions. Single samples showing a unique sequence variation, which was absent in any other sequence, were included in the Unassigned group (Un). In summary, 15 out of 20 sequences obtained in this study were located in Group 1, three samples were located in group 2 and two samples were located in Group 5. Groups 2 and 5 included only sequences obtained from this study, while sequences available in GenBank were located in the other Groups, mainly in Group 1 (*n* = 70) and in Group 10 (*n* = 10) (Table 4).

Analysis of the *ORF68* according to the grouping criteria previously proposed [4] showed that none of the sequences obtained in this study could be included in the six proposed groups, nor in the additional groups proposed later [7,10]. Indeed, in all samples a 118 bp nucleotide deletion was present at positions 701–818, resulting in a shorter amino acid sequence. The same deletion is present in KyA and Racl11 strains (MF975655 and MF975656), both isolated in the USA. *ORF68* sequences of Italian samples are very similar to sequences of KyA and Racl11, but these latter have a SNP at C236A, and KyA has further changes at T689G and T690C (Table 5). All *ORF68* sequences found in this study were identical and sample 09m142 represents all *ORF68* in Table 5.

## 4. Discussion

This study describes the sequence variations in important EHV-1 genes detected in archival samples of Italian horses and contributes to the knowledge of EHV-1 circulating in Italy. Unfortunately, limited data are available on Italian EHV-1 strains; only two sequences of the *ORF30* gene are deposited in GenBank (HM125711.1 and HM125712.1) and three strains have been recently investigated by a multi-locus sequence analysis approach [12], although sequences are not present in genetic databases. Two of these isolates (ITA/055/2011 and ITA/056/2011 were of the non-neuropathogenic type because they showed an adenine at position 2254 [12]. Three isolates (ITA/944/2003, 314102/BS/2009 and 16656/BS/2010) showed the substitution A2254G, that is considered a marker of the neuropathogenic genotype.

In this study we sequenced and deposited in GenBank sequences of *ORF30*, *ORF33*, *ORF34* and *ORF68* of EHV-1 detected in horses from three Italian Regions. A total of 18 out of 20 samples (90%) showed the mutation A2254G in the *ORF30*, confirming that N752D strains are common in outbreaks involving abortion. Although some samples included in this study were from the same stable, many of them were genetically different (*ORF33*, *ORF34*) and were considered distinct strains. The circulation of the N752D variant in Italy appears much higher than in other countries. Thirty-four out of 269 isolated in Ireland between 1990 and 2017 showed N752D [12], and only two out of 56 EHV-1 isolated from aborted fetuses in India had the neuropathogenic marker [8]. The N752D genotype was not found in EHV-1 isolated in Brazil [23], in Turkey [24] and in the 27 strains isolated in Poland between 1993 and 2017 [11]. Even in other countries the prevalence of the neuropathogenic genotype was rather low, as in Japan (2.7%), the USA (10.8–19.4%), Argentina (7.4%), France (24%), and Germany (10.6%) [25,26,27,28,29,30]. On the contrary, 90 out of 91 EHV-1 infecting equids in Ethiopia [9] and 12 out of 13 EHV-1 in Uruguay [31] had the variant N752D. Previous investigations in Italy showed that more than 60% of EHV-1 isolated mainly from aborted fetuses since 1980s in Italy possessed the mutation N752D in the *ORF30* [14], demonstrating that the hypervirulent type EHV-1 are spread in Italy since decades. Retrospective studies in the USA demonstrated that viruses with the neuropathogenic genotype increased from 3.3% in the 1960s to 14.4% in the 1990s and to 19.4% in the 2000–2006, suggesting that viruses with the neuropathogenic genotype are continuing to increase in prevalence within the latent reservoir of the virus [28]. Although vaccination remains the main tool for reducing viral spread and clinical disease, there is the evidence that vaccination against EHV-1 sometimes does not prevent abortion and spread of the neuropathogenic genotype [32]. In the present study abortion and spread of N752D strains were observed in vaccinated mares., but also in horses that are still unvaccinated, therefore, it is not surprising that the spread of type N752D in Italy has further increased. For this reason it is important to promote the application of corret vaccination programs in horses together with the research on new efficacious vaccines and new vaccination schedules.

The *ORF33* gene sequences obtained from some samples in this study were identical to the sequence of the reference strain Ab4 and to many other sequences present in GenBank [12,13,33]. Most samples showed the SNPs A1526T (N509I) and G2391A (synonymous), as reported in some EHV-1 from UK. Two samples showed a new SNP at position A1531G, corresponding to the amino acidic substitution N601D. Furthermore, sample 19m10 showed three new and non-synonymous changes at I810V, I838P and A861G. These data confirm that this sequence of the *ORF33* gene is generally highly conserved and it is a good target for diagnostic methods, although some SNPs were observed. Although a few single changes in a sequence usually do not affect the diagnostic sensitivity of standard or real-time PCR protocols, continuous monitoring of changes in this sequence are important to avoid false negative results due to viral mutations.

An extensive study on EHV-1 isolated in the UK demonstrated the highest sequence variability in the *ORF34* gene [13]. In the present study, some samples showed an *ORF34* sequence identical to the sequence of the reference strain Ab4. Other samples showed new SNPs that have not been reported before. A complete analysis of the *ORF34* sequences available in GenBank or reported in bibliography [12,13,33] showed that some SNPs are repeated in groups of strains. In the present study a method based on the comparison of SNPs was tentatively proposed for grouping different *ORF34* sequences. Twelve groups were found and named from 1 to 12. Group 1 includes the reference strain Ab4, Group 12 includes the strains isolated from zebra, onager and Thomson’s gazelle [34]. Sequences detected in the present study were located in Groups 1, 2 and 5. Most sequences available in GenBank were located in Group 1. Single strains with unique SNPs were provisionally included in the Unassigned group and will be included in a new group when other strains with the same SNPs will be found. Considering that limited investigations have been carried out so far, we can speculate that further SNPs will be found in the *ORF34* gene and that new groups will be described. Studies carried out on the *ORF34* product of EHV-1 suggest that the *ORF34* protein is required for optimal replication of EHV-1 in cultured cells at early times of infection [35]. The impact of different mutations in the *ORF34* gene on viral replication is not known.

Sequencing and analysis of the *ORF68* gene are widely used for grouping EHV-1 [4,7,10,11]. An extensive study on *ORF68* gene of EHV-1 isolated worldwide has been recently carried out [12]. After that six groups have been originally proposed [4], further SNPs have been described and new groups have been proposed [7,10,11]. All *ORF68* sequences detected in this study were not included in any group because they showed a 118 bp deletion in the nucleotide sequence 701–818 that have been not observed in any existing group. In particular, the sequence between nucleotides 1 and 700 was identical to those of the isolate IRL/268/2001 (MH976709.1), which was located in Group 4. However, members of Group 4 do not have the deletion found in this study, and it seems incorrect to locate our sequences in this group. The 118 bp deletion results in an amino acid sequence shorter than others and with a different sequence of the terminal 10 amino acids, resulting in unknown biological consequences. The same 118 bp deletion in the nucleotide sequence 701–818 is present in isolates RacL11 (MF975656.1) and KyA (MF975655.1). RacL11 has been isolated from an aborted foal and is more pathogenic than the attenuated Kentucky A (KyA), which is a candidate vaccine strain [36]. However, these isolates differ to our samples because they show also the variation C236A, that is not present in our samples.

## 5. Conclusions

This study describes the genetic characteristics of *ORF30*, *ORF33*, *ORF34* and *ORF68* genes of Italian EHV-1 detected in samples from horses with a history of abortion or recumbency. A very high prevalence of the N752D strains was found. Sequencing of *ORF33* gene confirmed the high conservation of this gene and showed few SNPs, some of which have not been previously reported. In this work a new efficient nPCR protocol to amplify the *ORF34* gene is described. Analysis of *ORF34* sequences obtained in this study and of those available in genetic databases showed new SNPs and suggested the existence of at least 12 different groups. Analysis of the *ORF68* sequences demonstrated an infrequent deletion of 118 bp in all Italian samples.

In conclusion, this study confirms the high variability of the *ORF34* gene and further investigations should assess whether this gene could be a useful marker for epidemiological studies. Furthermore, the presence of the 118 bp deletion in EHV-1 strains from other geographical areas and the pathogenic properties of isolates with this deletion should be evaluated.

## Figures and Tables

**Table 1:** Samples included in the study.

Code	Stable	Source	Vaccination
08m27	A	Organs	no
08m160	B	Organs	yes
09m34T	B	Organs	yes
09m45	C	NS	yes
09m68	D	NS	yes
09m142	E	NS	yes
09m209	E	NS	yes
09m217	F	NS	yes
10m01	G	Organs	no
10m106	J	Organs	yes
17m07	J	Organs	yes
17m13	K	Organs	yes
17m15	K	NS	yes
18m30	F	Organs	yes
19m04	E	Organs	no
19m05	C	Organs	no
19m08	L	CSF	no
19m10	M	Organs	yes
19m13	N	Organs	no
19m14	M	Organs	no

Organs were obtained by mixing lung and spleen samples from aborted fetuses; nasal swab (NS) samples were obtained from maresthat aborted; cerebrospinal fluid (CSF) sample was obtained from the recumbent horse 19m08. The year of collection is indicated by the numbers befor the “m” letter in the sample code (e.g., 09 means 2009, 19 means 2019). Stables from where the samples were collected are reported as alphabetical letters, no further information is provided to respect their privacy.

**Table 2:** Primers used for PCR and sequencing reactions.

Gene	Primer Name	Sequence (5′–3′)	Product Size (bp)	Annealing Temperature (°C)	Reference
*ORF30*	F8	GTGGACGGTACCCCGGAC	380	60	[18]
	R2	GTGGGGATTCGCGCCCTCACC			
	F7 *	GGGAGCAAAGGTTCTAGACC	256	60	[18]
	R3 *	AGCCAGTCGCGCAGCAAGATG			
*ORF33*	FC2	CTTGTGAGATCTAACCGCAC	1181	60	[17]
	RC	GGGTATAGAGCTTTCATGGG			
	FC2int *	CCGCACCTACGACCTAAAAA	940	58	This study
	RCint *	CGATCCCCTGCATAATCACT			
*ORF34*	1058F	GGCCCCAAGGATATTTAAGC	855	58	This study
	1893R	GTTTGAGGCGGTTACGTCAG			
	1090Fi *	CCGAGGTTTCATCCTCATTC	714	58	This study
	1784Ri *	GCGGACATATTCGTGTCTCA			
*ORF68*	68p1-Fe	AAGCATTGCCAAACAGTTCC	846	55	This study
	68p1-Re	CGAACACTCCCCAGAGTAGG			
	68p2-Fi *	TGAGCCGACAATGTTTCGTA	774	57	This study
	68p2-Ri *	GTTCCATCCACGTCACGTCT			

* Primers used in sequencing reactions.

**Table 3:** Nucleotide variations in *ORF33* gene of samples and of the reference isolate Ab4.

Code	*ORF33* SNPs					
	nt 1526	nt 1531	nt 2391	nt 2429	nt 2513	nt 2583
Ab4	A	A	G	A	A	C
08m27	A	G	G	-	-	-
08m160	unsp.					
09m34	T	A	A	A	A	C
09m45	T	A	A	A	A	C
09m68	T	A	A	-	-	-
09m142	T	A	A	A	A	C
09m209	unsp.					
09m217	unsp.					
10m01	A	G	G	-	-	-
10m106	T	A	A	-	-	-
17m07	-	-	-	-	-	-
17m13	T	A	A	-	-	-
17m15	T	A	A	-	-	-
18m30	-	-	-	-	-	-
19m04	-	-	-	-	-	-
19m05	-	-	-	-	-	-
19m08	-	-	-	-	-	-
19m10	A	A	G	G	C	G
19m13	-	-	-	-	-	-
19m14	-	-	-	-	-	-

Unsp. means that unspecific products have been obtained and sequenced; - means that negative results were obtained or that parts of sequences are missing. Samples 09m34, 09m45, 09m142 and 19m10 show longer sequences because visible products were obtained by first round PCR and good quality sequences were obtained.

**Table 4:** Variations of the 483 bp sequences of the *ORF34* gene are shown.

	Group	33	60	62	71	73	81	95	104	110	115	148	149	156	159	197	216	256	257	282	285	303	304	317	380	391	402	405	410	414	422	428	477	N
AY665713.1_Ab4	1	C	T	C	C	G	G	C	T	G	G	A	C	G	A	A	G	G	C	T	A	C	T	C	C	T	C	A	T	T	C	C	G	70
09m142	1	.	.	.	.	.	.	.	.	.	.	.	.	.	.	.	.	.	.	.	.	.	.	.	.	.	.	.	.	.	.	.	.	
09m209	2	.	C	.	.	.	.	.	.	.	.	.	.	.	.	.	.	.	.	.	.	.	.	.	.	.	.	.	.	.	.	.	.	0
09m34	2	.	C	.	.	.	.	.	.	.	.	.	.	.	.	.	.	.	.	.	.	.	.	.	.	.	.	.	.	.	.	.	.	
17m15	2	.	C	.	.	.	.	.	.	.	.	.	.	.	.	.	.	.	.	.	.	.	.	.	T *	.	.	.	C *	.	.	.	.	
KU206455.1_Buckin.93/2011	3	.	.	T *	.	.	.	.	.	.	.	.	.	.	.	.	.	.	.	.	.	.	.	.	.	.	.	.	.	.	.	.	.	5
KF644568.1_NMKT04	3	.	.	T *	.	.	.	.	.	.	.	.	.	.	.	.	.	.	.	.	.	.	.	.	.	.	.	.	.	.	.	.	.	
KF644579.1_89c25	4	.	.	.	.	A *	.	.	.	.	.	.	.	.	.	.	.	.	.	.	.	.	.	.	.	.	.	.	.	.	.	.	.	2
KF644577.1_89c105	4	.	.	.	.	A *	.	.	.	.	.	.	.	.	.	.	.	.	.	.	.	.	.	.	.	.	.	.	.	.	.	.	.	
19m04	5	.	.	.	.	.	.	.	.	.	.	.	T *	.	.	.	.	.	.	.	.	.	.	.	.	.	.	.	.	.	.	.	.	0
19m05	5	.	.	.	.	.	.	.	.	.	.	.	T *	.	.	.	.	.	.	.	.	.	.	.	.	.	.	.	.	.	.	.	.	
MF975655.1_KyA	6	.	.	.	.	.	.	.	.	.	.	.	.	T *	.	.	.	.	.	.	.	.	.	.	.	.	.	.	.	.	.	.	.	4
KF644570.1_NY05	6	.	.	.	.	.	.	.	.	.	.	.	.	T *	.	.	.	.	.	.	.	.	.	.	.	.	.	.	.	.	.	.	.	
KT324724.1_NZA-77	7	.	.	.	.	.	.	.	.	.	.	.	.	T *	.	.	.	.	.	.	.	A	.	.	.	.	.	.	.	.	.	.	.	6
KU206475.1_Lincoln.2/2012	7	.	.	.	.	.	.	.	.	.	.	.	.	T *	.	.	.	.	.	.	.	A	.	.	.	.	.	.	.	.	.	.	.	
KU206453.1_Kent/43/1994	8	.	.	.	.	.	.	.	.	.	.	.	.	.	.	G *	.	.	.	.	.	.	.	.	.	.	.	.	.	.	.	.	.	4
AY464052.1_V592	8	.	.	.	.	.	.	.	.	.	.	.	.	.	.	G *	.	.	.	.	.	.	.	.	.	.	.	.	.	.	.	.	.	
KF644578.1_01c1	9	.	.	.	.	.	.	.	.	.	.	.	.	.	.	.	.	.	T *	.	.	.	.	.	.	.	.	.	.	.	.	.	.	2
KF644566.1_90c16	9	.	.	.	.	.	.	.	.	.	.	.	.	.	.	.	.	.	T *	.	.	.	.	.	.	.	.	.	.	.	.	.	.	
KU206470.1_Oxfor.206/2013	10	.	.	.	.	.	.	.	.	.	.	.	.	.	.	.	.	.	.	.	C	.	.	.	.	.	.	G	.	.	.	.	.	10
KM593996.1_T953	10	.	.	.	.	.	.	.	.	.	.	.	.	.	.	.	.	.	.	.	C	.	.	.	.	.	.	G	.	.	.	.	.	
KT324733.1_717A-82	11	.	.	.	.	.	.	.	.	.	.	.	.	.	.	.	.	.	.	.	.	.	.	.	.	.	.	.	.	C	.	.	.	2
KT324725.1_3045-07	11	.	.	.	.	.	.	.	.	.	.	.	.	.	.	.	.	.	.	.	.	.	.	.	.	.	.	.	.	C	.	.	.	
KF644580.1_T-529_10/84	12	T	.	.	A *	.	A	.	C *	.	C *	G *	.	.	G	.	A	.	.	C	.	.	.	.	.	.	T	.	.	.	.	.	A	3
KF644575.1_94-137	12	T	.	.	A *	.	A	.	C *	.	C *	G *	.	.	G	.	A	.	.	C	.	.	.	.	.	.	T	.	.	.	.	.	A	
KF644574.1_T-616	12	T	.	.	A *	.	A	.	C *	.	C *	G *	.	.	G	.	A	.	.	C	.	.	.	.	.	.	T	.	.	.	.	.	A	
AP012321.1_5586	Un1	.	.	.	.	.	.	T *	.	A *	.	.	.	.	.	.	.	.	.	.	.	.	.	.	.	.	.	.	.	.	.	T *	.	6
KU206432.1_Cambrid..3/1995	Un2	.	.	.	.	.	.	.	.	.	.	.	.	.	.	.	.	C *	.	.	.	.	.	.	.	.	.	.	.	.	.	.	.	
KF644576.1_00c19	Un3	.	.	.	.	.	.	.	.	.	.	.	.	.	.	.	.	.	.	.	.	.	C *	.	.	.	.	.	.	.	.	.	.	
KY852346.1_Hertf.150/2016	Un4	.	.	.	.	.	.	.	.	.	.	.	.	.	.	.	.	.	.	.	.	.	.	T *	.	.	.	.	.	.	.	.	.	
MF975656.1_RacL11	Un5	.	.	.	.	.	.	.	.	.	.	.	.	.	.	.	.	.	.	.	.	.	.	.	.	C *	.	.	.	.	.	.	.	
KU206462.1_Hampsh.1/2008	Un6	.	.	.	.	.	.	.	.	.	.	.	.	.	.	.	.	.	.	.	.	.	.	.	.	.	.	.	.	.	T *	.	.	

Variations marked with an asterisk (*) are nonsynonymous point mutations, which changes the single nucleotide into a codon that does not translate into the same amino acid; mutations without an asterisk are synonymous mutations. Dots (.) indicate sequence identity. Only representative samples obtained in this study or representative sequences of the 114 *ORF34* sequences available in GenBank are shown in each group with similar type of mutations; sequences identical to the selected representative sequences were not included in the table. A group was generated when at least two samples or database sequences showed the same variations at the same positions. Single samples with a particular sequence variation were included in the Unassigned group (Un). Six different unassigned groups were found and named from Un1 to Un6. Column N indicates the number of sequences present in GenBank (last access 21st July 2019) and showing nucleotide variations typical of each specific group. The isolate Buckinghamshire/9/93 was tentatively included in Group 1 although it had a frameshift caused by a deletion of nucleotides 231–232 [13].

**Table 5:** Nucleotide variations in *ORF68* of samples and of isolates grouped according to Nugent et al., 2006 [4].

	Group	236	336	344	620	626	629	689–690	701	710	713	719	738–739	743	755	783	818	821	825
AY665713.1_Ab4	1	C	C	G	C	T	G	TT	G	T	C	G	GG	C	C	G	G	G	C
DQ172400.1_US85_1_1	1	*	.	.		.	.	..	.	.	.	.	GGG	.	.	.	.	.	.
DQ172310.1_AR85_1_1	1	*	.	.		.	.	..	.	.	.	.	..	.	.	.	.	.	.
MH976707.1_IRL/559/2009	2	.	.	.		.	.	..	.	.	.	.	..	.	.	.	.	.	.
DQ172408.1_US89_1_1	2	*	.	.	.	.	.	..	.	.	.	.	..	T	.	.	.	.	.
DQ172394.1_US79_1_1	2	*	.	.	T	.	.	..	.	.	.	.	..	.	.	.	.	.	G
DQ172309.1_AR79_1_1	2	*	.	.	.	.	.	..	.	C	.	.	..	.	.	.	.	.	.
DQ172384.1_US03_5_2	2	*	.	.	.	.	.	..	.	.	.	.	..	.	.	T	.	.	.
MH976708.1_IRL/471/2008	3	.	.	.	.	.	A	..	.	.		T	..	.	.	.	.	.	.
MH976706.1_IRL/307/2015	3	.	.	.	.	.	A	..	.	.	.	T	..	.	.	.	.	A	.
DQ172365.1_GB89_2_1	3	*	.	.	.	.	A	..	.	A	.	T	..	.	.	.	.	.	.
MH976709.1_IRL/268/2001	4	.	.	.	.	.	A	..	.	.	.	.	..	.	.	.	.	.	.
DQ172332.1_GB00_1_1	4	*	.	A	.	.	A	..	.	.	.	.	..	.	.	.	.	.	.
MH976705.1_IRL/837/2007	5	.	.	.	.	C	A	..	.	G	A	.	..	.	.	.	.	.	.
DQ172375.1_US01_1_2	5	*	.	.	.	.	A	..	.	G	A	.	..	.	.	.	.	.	.
AY464052.1_V592	6	.	T	.	.	.	A	..	.	.	.	.	..	.	T	.	.	.	.
MH976703.1_IRL/069/1995	6	.	T	.	.	.	A	..	.	.	.	.	..	.	T	.	.	.	.
DQ172359.1_GB85_1_1	6	*	T	.	.	.	A	..	.	.	.	.	..	.	T	.	.	.	.
09m142		.	.	.	.	.	A	..	Start gap	-	-	-	-	-	-	-	End gap	.	.
MF975656.1_RacL11		A	.	.	.	.	A	..	Start gap	-	-	-	-	-	-	-	End gap	.	.
MF975655.1_KyA		A	.	.	.	.	A	GC	Start gap	-	-	-	-	-	-	-	End gap	.	.

Sequences are aligned with reference to the prototype isolate Ab4 (AY665713.1). Dashes (-) indicate gaps in the alignment, dots (. or ..) indicate sequence identity, asterisks (*) indicate nucleotides not available in GenBank or ENA. Sample 09m142 represents all *ORF68* sequences found in this study, which show identical sequences. EHV-1 RacL11 and KyA and all EHV-1 detected in this study showed a 118 bp deletion between nucleotide positions 701 and 818, resulting in the deletion of a sequence of 40 amino acids.

## Supplementary Materials

The following are available online at https://www.mdpi.com/1999-4915/11/9/851/s1, Table S1: GenBank Accession Numbers of selected sequences obtained in this study.

## References

[1] OIE-Listed Diseases, Infections and Infestations in Force in 2019. www.oie.int.

[2] Telford E.A., Watson M.S., McBride K., Davison A.J. (1992). The DNA sequence of equine herpesvirus-1. Virology.

[3] OIE (World Organization for Animal Health) (2017). Chapter 2.5.9: Equine rhinopneumonitis (infection with equid herpesvirus-1 and -4). OIE Terrestrial Manual.

[4] Nugent J., Birch-Machin I., Smith K.C., Mumford J.A., Swann Z., Newton J.R., Bowden R.J., Allen G.P., Davis-Poynter N. (2006). Analysis of Equid Herpesvirus 1 Strain Variation Reveals a Point Mutation of the DNA Polymerase Strongly Associated with Neuropathogenic versus Nonneuropathogenic Disease Outbreaks. J. Virol..

[5] Allen G.P., Breathnach C.C. (2006). Quantification by real-time PCR of the magnitude and duration of leucocyte-associated viraemia in horses infected with neuropathogenic vs. non-neuropathogenic strains of EHV-1. Equine Vet. J..

[6] Goodman L.B., Loregian A., Perkins G.A., Nugent J., Buckles E.L., Mercorelli B., Kydd J.H., Palù G., Smith K.C., Osterrieder N. (2007). A Point Mutation in a Herpesvirus Polymerase Determines Neuropathogenicity. PLoS Pathog..

[7] Malik P., Balint A., Dan A., Palfi V. (2012). Molecular characterisation of the *ORF68* region of equine herpesvirus-1 strains isolated from aborted fetuses in Hungary between 1977 and 2008. Acta Vet. Hung..

[8] Anagha G., Gulati B.R., Riyesh T., Virmani N. (2017). Genetic characterization of equine herpesvirus 1 isolates from abortion outbreaks in India. Arch. Virol..

[9] Negussie H., Gizaw D., Tessema T.S., Nauwynck H.J. (2017). Equine Herpesvirus-1 Myeloencephalopathy, an Emerging Threat of Working Equids in Ethiopia. Transbound Emerg. Dis..

[10] Stasiak K., Dunowska M., Hills S.F., Rola J. (2017). Genetic characterization of equid herpesvirus type 1 from cases of abortion in Poland. Arch. Virol..

[11] Matczuk A.K., Skarbek M., Jackulak N.A., Bazanow B.A. (2018). Molecular characterisation of equid alphaherpesvirus 1 strains isolated from aborted fetuses in Poland. Virol. J..

[12] Garvey M., Lyons R., Hector R.D., Walsh C., Arkins S., Cullinane A. (2019). Molecular Characterisation of Equine Herpesvirus 1 Isolates from Cases of Abortion, Respiratory and Neurological Disease in Ireland between 1990 and 2017. Pathogens.

[13] Bryant N.A., Wilkie G.S., Russell C.A., Compston L., Grafham D., Clissold L., McLay K., Medcalf L., Newton R., Davison A.J. (2018). Genetic diversity of equine herpesvirus 1 isolated from neurological, abortigenic and respiratory disease outbreaks. Transbound Emerg. Dis..

[14] Autorino G.L., Corradi V., Frontoso R., Galletti S., Manna G., Mascioni A., Pallone A., Ricci I., Rosone F., Simula M., Delogu R., Falcone E., Monini M., Ruggeri F.M., Di Martino B., Marsilio F., Monaco F., Savini G. (2014). P.3 Gestione di un focolaio neurologico da Equine herpesvirus 1 (EHV-1). Workshop Nazionale di Virologia Veterinaria.

[15] Preziuso S., Cuteri V. (2012). A Multiplex Polymerase Chain Reaction Assay for Direct Detection and Differentiation of β-Hemolytic Streptococci in Clinical Samples from Horses. J. Equine Vet. Sci..

[16] Preziuso S., Pinho M.D., Attili A.R., Melo-Cristino J., Acke E., Midwinter A.C., Cuteri V., Ramirez M. (2014). PCR based differentiation between Streptococcus dysgalactiae subsp. equisimilis strains isolated from humans and horses. Comp. Immunol. Microbiol. Infect. Dis..

[17] Wang L., Raidal S.L., Pizzirani A., Wilcox G.E. (2007). Detection of respiratory herpesviruses in foals and adult horses determined by nested multiplex PCR. Vet. Microbiol..

[18] Allen G.P. (2006). Antemortem detection of latent infection with neuropathogenic strains of equine herpesvirus-1 in horses. Am. J. Vet. Res..

[19] Untergasser A., Nijveen H., Rao X., Bisseling T., Geurts R., Leunissen J.A.M. (2007). Primer3Plus, an enhanced web interface to Primer3. Nucleic Acids Res..

[20] Hall T.A. (1999). BioEdit: A user-friendly biological sequence alignment editor and analysis program for Windows 95/98/NT. Nucl. Acids Symp. Ser..

[21] Edgar R.C. (2004). MUSCLE: Multiple sequence alignment with high accuracy and high throughput. Nucleic Acids Res..

[22] Kumar S., Stecher G., Tamura K. (2016). MEGA7: Molecular Evolutionary Genetics Analysis Version 7.0 for Bigger Datasets. Mol. Biol. Evol..

[23] Mori E., Lara d.C.C.S.H., Cunha E.M.S., Villalobos E.M.C., Mori C.M.C., Soares R.M., Brandao P.E., Fernandes W.R., Richtzenhain L.J. (2015). Molecular characterization of Brazilian equid herpesvirus type 1 strains based on neuropathogenicity markers. Braz. J. Microbiol..

[24] Turan N., Yildirim F., Altan E., Sennazli G., Gurel A., Diallo I., Yilmaz H. (2012). Molecular and pathological investigations of EHV-1 and EHV-4 infections in horses in Turkey. Res. Vet. Sci..

[25] Perkins G.A., Goodman L.B., Tsujimura K., Van de Walle G.R., Kim S.G., Dubovi E.J., Osterrieder N. (2009). Investigation of the prevalence of neurologic equine herpes virus type 1 (EHV-1) in a 23-year retrospective analysis (1984–2007). Vet. Microbiol..

[26] Vissani M.A., Becerra M.L., Olguín Perglione C., Tordoya M.S., Miño S., Barrandeguy M. (2009). Neuropathogenic and non-neuropathogenic genotypes of Equid Herpesvirus type 1 in Argentina. Vet. Microbiol..

[27] Pronost S., Leon A., Legrand L., Fortier C., Miszczak F., Freymuth F., Fortier G. (2010). Neuropathogenic and non-neuropathogenic variants of equine herpesvirus 1 in France. Vet. Microbiol..

[28] Smith K.L., Allen G.P., Branscum A.J., Frank Cook R., Vickers M.L., Timoney P.J., Balasuriya U.B.R. (2010). The increased prevalence of neuropathogenic strains of EHV-1 in equine abortions. Vet. Microbiol..

[29] Fritsche A.K., Borchers K. (2011). Detection of neuropathogenic strains of Equid Herpesvirus 1 (EHV-1) associated with abortions in Germany. Vet. Microbiol..

[30] Tsujimura K., Oyama T., Katayama Y., Muranaka M., Bannai H., Nemoto M., Yamanaka T., Kondo T., Kato M., Matsumura T. (2011). Prevalence of equine herpesvirus type 1 strains of neuropathogenic genotype in a major breeding area of Japan. J. Vet. Med. Sci..

[31] Castro E.R., Arbiza J. (2017). Detection and genotyping of equid herpesvirus 1 in Uruguay. Rev. Sci. Tech..

[32] Damiani A.M., de Vries M., Reimers G., Winkler S., Osterrieder N. (2014). A severe equine herpesvirus type 1 (EHV-1) abortion outbreak caused by a neuropathogenic strain at a breeding farm in northern Germany. Vet. Microbiol..

[33] Vaz P.K., Horsington J., Hartley C.A., Browning G.F., Ficorilli N.P., Studdert M.J., Gilkerson J.R., Devlin J.M. (2016). Evidence of widespread natural recombination among field isolates of equine herpesvirus 4 but not among field isolates of equine herpesvirus 1. J. Gen. Virol..

[34] Guo X., Izume S., Okada A., Ohya K., Kimura T., Fukushi H. (2014). Full genome sequences of zebra-borne equine herpesvirus type 1 isolated from zebra, onager and Thomson’s gazelle. J. Vet. Med. Sci..

[35] Said A., Damiani A., Osterrieder N. (2014). Ubiquitination and degradation of the *ORF34* gene product of equine herpesvirus type 1 (EHV-1) at late times of infection. Virology.

[36] Shakya A.K., O’Callaghan D.J., Kim S.K. (2017). Comparative Genomic Sequencing and Pathogenic Properties of Equine Herpesvirus 1 KyA and RacL11. Front. Vet. Sci..

