# Supplementary material for: Equid alphaherpesvirus 1 from Italian Horses: Evaluation of the Variability of the ORF30, ORF33, ORF34 and ORF68 Genes
Source: Viruses. 2019 Sep 13;11(9):851. doi: 10.3390/v11090851 (PMC6784080; doi:10.3390/v11090851)
Supplement: Supplementary file 1 [file viruses-11-00851-s001.pdf]

Supplementary Table 1: GenBank Accession Numbers of selected sequences obtained in this study. Only 23 out of 57 sequences obtained were deposited in order to avoid that too many identical sequences were included in GenBank. Sequences obtained but not deposited in GenBank because identical to other deposited sequences are indicated by the abbreviation "Idt" followed by the GenBank Accession Number of the deposited identical sequence. (-) means that PCR products for sequencing or sequences were not obtained or that low quality sequences were obtained.

Code	GenBank Accession Number			
	**ORF30**	**ORF33**	**ORF34**	**ORF68**
**08m27**	Idt MN226968	MN226970	Idt MN226976	MN226985
**08m160**	Idt MN226969	-	MN226974	-
**09m34**	Idt MN226969	Idt MN226971	MN226975	-
**09m45**	Idt MN226969	MN226971	Idt MN226976	MN226986
**09m68**	Idt MN226969	Idt MN226971	Idt MN226976	-
**09m142**	Idt MN226969	MN226972	MN226976	MN226987
**09m209**	MN226969	-	MN226977	MN226988
**09m217**	Idt MN226969	-	MN226978	-
**10m01**	Idt MN226969	Idt MN226970	Idt MN226976	Idt MN226987
**10m106**	Idt MN226969	Idt MN226971	MN226979	-
**17m07**	Idt MN226969	-	Idt MN226976	-
**17m13**	Idt MN226969	Idt MN226971	MN226980	-
**17m15**	Idt MN226969	Idt MN226971	MN226981	-
**18m30**	Idt MN226969	-	MN226982	MN226989
**19m04**	Idt MN226969	-	MN226983	-
**19m05**	Idt MN226969	-	MN226984	-
**19m08**	Idt MN226969	-	Idt MN226976	-
**19m10**	MN226968	MN226973	Idt MN226976	MN226990
**19m13**	Idt MN226969	-	Idt MN226976	-
**19m14**	Idt MN226969	-	Idt MN226976	-
